# Television Viewing Time is Associated With Overweight/Obesity Among Older Adults, Independent of Meeting Physical Activity and Health Guidelines *Reply to Stabler and Colleagues*

**DOI:** 10.2188/jea.JE20130079

**Published:** 2013-09-05

**Authors:** Shigeru Inoue, Takemi Sugiyama, Tomoko Takamiya, Koichiro Oka, Neville Owen, Teruichi Shimomitsu

**Affiliations:** 1 Department of Preventive Medicine and Public Health, Tokyo Medical University, Tokyo, Japan; 2 Baker IDI Heart and Diabetes Institute, Melbourne, Australia; 3 Faculty of Sport Sciences, Waseda University, Tokorozawa, Saitama, Japan; 4 Cancer Prevention Research Centre, School of Population Health, The University of Queensland, Brisbane, Australia

**In Reply:** We thank Stabler and colleagues for their valuable comments^1^ on our work. We agree that the analytical approach used in our study does not necessarily support the conclusion that TV viewing time is associated with overweight/obesity, independent of moderate-to-vigorous physical activity (MVPA). Our intention was to examine whether TV viewing time was detrimentally associated with adiposity even among those who met physical activity guidelines. However, as Stabler and colleagues point out, it is misleading to use the term “independent” to characterize these associations, given how we originally approached our analyses. It is also appropriate to query our rather colloquial use of the term “risk”. Strictly speaking, “odds” is what is statistically and epidemiologically correct for a cross-sectional study using logistic regression.

Following this helpful advice, we conducted an additional analysis in which TV viewing time, MVPA, and their interaction term were included (along with the same covariates used in the original adjusted models). The TV time/MVPA interaction term was found to be nonsignificant (*P* = 0.408), which suggests that the relationship between TV time and the odds of being overweight or obese is not significantly moderated by MVPA levels and can be interpreted as consistent with our original conclusion. Nevertheless, we must agree that the analytic approach recommended by Stabler and colleagues is formally correct and appropriate in this case. We appreciate their thoughtful attention to our work and are grateful for the opportunity to re-examine our data and more appropriately state our conclusion.
